# Healthcare-associated respiratory viral infections are an important cause of mortality and morbidity– evidence from cohort study of 1700 patients

**DOI:** 10.1017/ash.2025.142

**Published:** 2025-09-03

**Authors:** Zongxin Dai, Paul Anantharajah Tambyah, Somani Jyoti, Hwang Ching Chan, Revathi Sridhar, Vennila Gopal, Sean Wu

**Affiliations:** 1 National University Hospital, Singapore, Department of Medicine, Division of Infectious Diseases; 2 National University of Singapore, Yong Loo Lin School of Medicine

## Abstract

**Objectives:** Healthcare-associated respiratory viral infections (HA-RVI) remain a threat despite the fading memory of the pandemic. To better understand the impact of HA-RVI, we reviewed endemic respiratory viral infections in our tertiary academic hospital just before the pandemic. **Methods:** A retrospective analysis of a hospital epidemiology database with all patients tested positive for respiratory viruses between Jan2016 – Dec2019 was conducted. Testing was ordered by attending physicians and done using immunofluorescence assays or multiplex PCR. HA-RVI patients were identified based on positive virologic tests >48 hours after admission. Data analyses were performed on Vassar Stats. **Results:** Of the 1700 patients included in this study, 315(18.5%) had HA-RVI while 1385(81.5%) had community-acquired infections (CAI). Influenza, enterovirus/rhinoviruses and respiratory syncytial virus were the most common viruses. Compared with CAI, HA-RVI patients were older (mean age 37.1±29.7 vs 21.4±27.5 years, p<0.001), had recent hospitalisations (OR 1.5, 95% CI:1.1-2.1, p=0.007), underlying bronchial asthma or COPD (OR 2.8, 95% CI:1.5-5.3, p=0.002) and were immunosuppressed (OR 7.6, 95% CI:4.3-13.4, p<0.001). Interestingly, HA-RVI patients were less likely to have fever (49.8% vs 66.4%, p<0.001), cough (42.2% vs 67.3%, p<0.001) or shortness of breath (17.5% vs 26.6%, p=0.02). Despite fewer symptoms, HA-RVI patients were more likely to have pneumonia with abnormal chest x-rays (33.3% vs 22.7%, RR 1.14, 95% CI:1.02-1.29, p=0.04), longer lengths of stay (mean 21.2±36.7 vs 5±14 days, p<0.001), higher rates of ICU admission (14.9% vs 8.1%, OR 2.0, 95% CI:1.4-2.9, p<0.001), and mortality (4.8% vs 0.6%, OR 8.6, 95% CI:3.6-20.5, p<0.001). **Conclusions:** Patients who are older, have pre-existing respiratory disorders or are immunosuppressed face greater HA-RVI risk. HA-RVI patients are less likely to exhibit typical respiratory infection symptoms, potentially delaying diagnosis. This probably contributes to increased morbidity and mortality associated with HA-RVI which underscore the importance of hospital infection prevention even for endemic respiratory viruses.

